# Exploring the Predictability of Temperatures in a Scaled Model of a Smarthome

**DOI:** 10.3390/s21186052

**Published:** 2021-09-09

**Authors:** Thomas Burns, Gregory Fichthorn, Jason Ling, Sharare Zehtabian, Salih S. Bacanlı, Ladislau Bölöni, Damla Turgut

**Affiliations:** 1 Department of Computer Science, Rutgers University, Piscataway, NJ 08854, USA; tjburns28@gmail.com; 2 Department of Computer Science, Stetson University, DeLand, FL 32723, USA; gfichthorn@stetson.edu; 3 Department of Computer Science, Pennsylvania State University, State College, PA 16802, USA; jling5242@gmail.com; 4 Department of Computer Science, University of Central Florida, Orlando, FL 32816, USA; sharare.zehtabian@knights.ucf.edu (S.Z.); bacanli@knights.ucf.edu (S.S.B.); ladislau.boloni@ucf.edu (L.B.)

**Keywords:** smart home, scaled model, machine learning, temperature prediction

## Abstract

In modern smarthomes, temperature regulation is achieved through a mix of traditional and emergent technologies including air conditioning, heating, intelligent utilization of the effects of sun, wind, and shade as well as using stored heat and cold. To achieve the desired comfort for the inhabitants while minimizing environmental impact and cost, the home controller must predict how its actions will impact the temperature and other environmental factors in various parts of the home. The question we are investigating in this paper is whether the temperature values in different rooms in a home are predictable based on readings from sensors in the home. We are also interested in whether increased accuracy can be achieved by adding sensors to capture the state of doors and windows of the given room and/or the whole home, and what type of machine learning algorithms can take advantage of the additional information. As experimentation on real-world homes is highly expensive, we use ScaledHome, a 1:12 scale, IoT-enabled model of a smart home for data acquisition. Our experiments show that while additional data can improve the accuracy of the prediction, the type of machine learning models needs to be carefully adapted to the number of data features available.

## 1. Introduction

In modern smarthomes, temperature regulation is achieved through a mix of traditional and emergent technologies including air conditioning, heating, intelligent utilization of the effects of sun, wind, and shade as well as using stored heat and cold. Modern internet of things (IoT) technology allows many of the actuators affecting the state of the home to be controllable at a distance—these include air conditioning and heating units, remote-controlled variable vents and automatically actuated indoor and outdoor shades. While remote-controlled doors and windows are rarely used in private homes, the technology exists and is widely deployed in office and industrial settings. For this paper, we assume that all the actuators affecting the thermal comfort of the home are IoT controlled. We will refer to as the *home controller* the control logic that autonomously controls these devices.

The objective of the home controller is to strike an optimal balance between the comfort of the inhabitants and the minimization of the environmental impact and cost. Many currently deployed controllers such as the Nest thermostat series from Google implement a human-programmed, if-then-else type logic coupled with presence detectors and timers. However, such manual programming gets increasingly harder and more error-prone with the increase in the number of sensors and actuators. While some of these systems are marketed as “learning systems”, the learning component usually comprises a limited aspect of the functionality, such as learning when the users typically leave to and return from work.

How can we create a fully autonomous home controller that achieves the desired comfort level while minimizing cost and environmental impact? Let us assume that the set of temperatures in the *m* different rooms of the home are a vector $t=\{t_{1},\ldots ,t_{m}\}$. At any timestep, the home controller will need to take a package of actions corresponding to the *n* actuators $a=\{a_{1},a_{2},\ldots ,a_{n}\}$. A convenient, rigorous approach for choosing the actions is based on *model predictive control* (MPC): if we have a model that can *predict* the effects of the actions one or several timesteps away, we can choose the actions that optimize comfort while minimizing costs.

How can we create such a prediction model? A predictor built from first principles must take into consideration the radiant heat from the sun, how the solar radiation is reduced by external obstacles such as trees and by internal obstacles such as shades, the light absorption properties of the roof and walls, the thermal isolation properties of the walls, the airflow in the building considering the open or closed states of the doors and windows, the pressure in the heating and air conditioning pipes as well as many other factors. Furthermore, these factors interact with each other, leading to a further increase in complexity. Performing such an analysis for a typical suburban home is unfeasible.

The question we investigate in this paper is whether it is possible to *learn* such a predictive model. One of the challenges is that the amount of data that can be realistically collected from a home before we need to deploy the system is limited. Let us consider that the amount of the observations we can afford to have is $\left\{o_{1},\ldots ,o_{k}\right\}$, where an observation $o_{i}$ is a collection of actions and measurements: $o_{i}=\langle \left\{a_{ji}\right\},\left\{m_{ki}\right\}\rangle$. Before we commit to deploying a learning-based system, we need to know whether this learning is possible. That is, is it possible to train a predictor $P\leftarrow P(\left\{o_{i}\right\})$ that, given a current set of measurements $\left\{m_{kt}\right\}$ and a series of future actions $a_{t+1},\ldots ,a_{t+\Delta t}$ will return a prediction of measurements ${\hat{m}}_{t+1}\ldots {\hat{m}}_{t+\Delta t}$? Clearly, the model will be specific to a given home. For instance, even two identical homes constructed face to face on a street would have a different model, as the north-south orientation of the windows and rooms would be reversed. The position of trees, the color of the house, and the tiles would also change the model. Thus, the question we are interested in is not to learn a model offline which can be then applied to any home—this is not possible. We can, however, answer the question of whether such a model is *learnable*, how much data we need to learn it, how many sensors do we need to deploy and what machine learning techniques would be for a specific type of sensing data.

As experimentation on real-world homes under various experimental conditions is prohibitively expensive, a possible solution might be to use a simulated model of the home. However, building a realistic simulation that takes into consideration all the environmental factors and their interactions is the same unfeasible problem as building a predictive model from first principles. While high-performance building energy modeling programs such as EnergyPlus [1] can accurately model a building provided that precise information is available, such modeling requires time, information, and expertise which is only cost-effective for commercial buildings.

Fortunately, in order to answer the learnability question, the primary thing we need to get right is the dependency matrix between the environmental features, actions, and measurements. Which actions influence which measurements? Which previous measurements the new measurement depends on? Getting the exact parameters right is less important because these parameters will anyhow vary between the real-world homes.

Thus, in this paper, we will use the ScaledHome testbed, a *scaled physical model* of a suburban home to collect experimental data. We want to emphasize that the exact temperatures and other measurements will not be the same as in a full-scale home. This is partially due to the difficulties of collecting data about the real home at a high enough level of detail, a difficulty shared with the simulator-based modeling approaches. In addition, scaled models also present another difficulty, due to the dimensionality: the 1:12 scale ScaledHome has 1/144th of the surfaces but only 1/1728th of the air volume of the original home. Due to this dimensionality problem, it is essentially impossible to simultaneously match the measured temperatures and other environmental factors without a complex, nonlinear mapping function.

The research objective of this paper is to develop a method through which we can identify whether, for a given home and a set of sensors, it is possible to train a machine learning model that can predict the temperatures in rooms. Furthermore, we are interested to know what machine learning algorithms and with what parameters, achieve the best performance. Our objective is not the exact modeling. For instance, we do not aim to train the home controller on the model before the home is built. What we argue that the home controller must be trained on data collected from the real home operating in its natural environment. By implementing the appropriate layouts of rooms, measurement points, actuators, and external environment, ScaledHome faithfully models the existence and general direction of *dependencies* between these values. Thus, we can have high confidence that a machine learning model that learns to predict in the scaled home, can do the same in a physical home, requiring a similar amount of training data and yielding similar accuracy. What is going to be transferred from the scaled home to the real home is the learning approach and the algorithms; the machine learning model itself will need to be re-learned with real-world data.

## 2. Related Work

### 2.1. Scale Models

The ScaledHome project fits in the multi-decade tradition of scaled models of physical phenomena. Such models had been traditionally used whenever the modeled system is too complex for all its functionality to be expressed as a closed-form formula, but the building of full-scale models is expensive or impractical [2]. Translating the observations from the scaled model to the real world (and back) has been known as the similitude problem in fields such as aerospace engineering, vehicle design, heating, and cooling as well as structural engineering [3,4]. The theoretical justification behind these models is the Buckingham π theorem, the formalization of the Rayleigh method. The intuition is that any equation describing a physical system with *n* variables can be described in terms of p=n−k dimensionless parameters where *k* is the number of physical dimensions. These dimensionless parameters will be used to formulate scaling laws for the model and real-world phenomena.

The applicability of scale models to air conditioning was shown as early as the 1980s and 1990s in the work of Olson [5] and Okutan [6]. Awbi and Nemri [7] performed a numerical modeling study whose output can be used to model the physical movement of air under complex thermal conditions. Nagano et al. [8] used a scale model to study the properties of a proposed air conditioning approach using a granular phase-change material.

The scaling down of the heat flow of a data center was also studied in [9], with mixed results—while for the temperature distribution the error was low (3.7% and 14.7%), the velocity and flow patterns were not accurate. Nada et al. [10] studied the thermal management in a data center using a scaled physical model, and uses the model to investigate the energy efficiency of different air distribution systems.

Mendez and Ordóñez [11] describe an algorithm that obtains the scaling laws in a mechanical structure in the form of a power-law derived from experimental data (including simulated experiments). Shehadeh et al. [12] used physical models for large structural elements, such as the components of petroleum oil drilling rigs. Zapico et al. [13] describe a technique to update a 1:50 scale model of a multi-span motorway bridge used to estimate the natural frequencies of the bridge. Another study of dynamic testing of structures is described by Jha et al. [14].

In a different type of application, Wosnik et al. [15] used a scaled physical model to perform the hydraulic analysis necessary for the design of an auxiliary spillway for an existing flood control dam at Canton, Oklahoma.

### 2.2. Temperature and Energy Management in Smart Homes

In the last decade, a number of academic projects developed smart homes that served as a testbed for various IoT technologies that are currently seeing gradual commercial adoption. Such systems included the MavHome smart home [16], the Gator Tech smart home [17] and the CASAS project [18]. Beyond technology demonstrations, these systems often yielded publicly accessible databases. Data collected from real-world homes with a representative population of inhabitants such as the Smart* dataset [19] will always remain the gold standard for modeling accuracy. Nothing can model the impacts of a Florida Summer or Minnesota Winter better than building actual homes in those locations, connecting them to the local utilities and energy trading networks, populating them with real people, and collecting data over the course of years. Such experimentation, however, is slow and costly in terms of human and financial resources.

Mateo et al. [20] studied the use of machine learning techniques to predict the temperature in different rooms of a home. The techniques proposed included extreme learning machines, combinations of multi-layer perceptrons with non-linear autoregressive techniques which were compared to baseline techniques such as multiple linear regression and autoregressive exogenous models. The data had been obtained from a 3D building modeling simulator.

In many applications, the goal of the predictive model is to serve as a component of an efficient smart home controller that automatically maintains the comfort levels of the inhabitants. Jin et al. [21] uses a predictive model of temperature values to create a controller that maintains the comfort of the occupants of a home, while simultaneously optimizing the energy usage. The predictive model is based on an LSTM model trained on recorded values of indoor and outdoor temperature and humidity values. The authors show that the controller can achieve comfort levels with approximately 8% lower energy consumption.

Another research direction in smart energy solutions concerns the integration of the various components including sensing, demand response, load control, and pricing. One of the earliest papers that described an integrated solution for these is by Han and Lim [22] which employed IEEE 802.15.4 and ZigBee to connect the components of the smart home into an integrated system. Cvitić et al. [23] provides a novel classification of IoT devices in smart home environments based on the features of the traffic flow and in [24] describe how to learn to classify using logistic regression and the logitboost method.

A significant number of recent research papers aim to develop machine learning models for the prediction of temperature values in different zones of a home. Irshad et al. [25] use a small neural network trained with the Levenberg-Marquardt algorithm to predict the temperature and the thermal comfort of the occupants of a room fitted with novel air conditioning system. The system takes into account factors such as the solar radiation, clothing and metabolic rate of experimental subjects. The experiment had been performed in a tropical climate region in Malaysia. Verma, Prakash and Kumar [26] describe a multi-agent system-based controller for an energy efficient building, that aims to minimize the difference between the consumer preferences and the measured values for temperature, light and CO${}_{2}$ values. Zamora-Martínez et al. [27] describes an approach to predict temperatures in the SML house, an experimental solar house designed to improve energy efficiency. The system uses both single step and multi-step forecasting. The algorithms investigated are auto-regressive moving average (ARIMA) variations as well as a single layer neural network in a multi-input multi-output setup. A follow-up paper [28] also extends the estimation techniques to Bayesian estimation.

A research group including Spencer, Alfandi and Al-Obeidat use data from the same home to perform predictive modeling using forward stepwise linear resolution [29], variants of lasso regression [30] and ridge regression [31]. Kumar, Pal and Singh [32] propose several approaches based on particle swarm optimization, extreme learning machine (ELM) and a variation called online sequential ELM. The proposed approaches are also evaluated on the SLM house dataset. Potočnik, Primož et al. [33] are also predicting the short-term variations of temperature of buildings using neural networks, autoregressive models with exogenous inputs and extreme learning machines. The home considered in this case is a residential building heated by a heat pump in a typical winter in Slovenia.

While the papers considered until now are directly aiming to predict temperature values, another research direction considers the related concept of the thermal comfort of the inhabitants. Magalhães, Leal and Horta [34] are considering the relationship between the heating energy and thermal comfort, while also taking into consideration the behavior of the inhabitants. Escandón et al. [35] is using an artificial network to predict the thermal comfort. The authors used as input data EnergyPlus-based simulations of generic models of the typical housing stock built in the post-war period in Southern Europe.

## 3. Data Collection

### 3.1. The ScaledHome-2 Testbed

**Figure 1:**
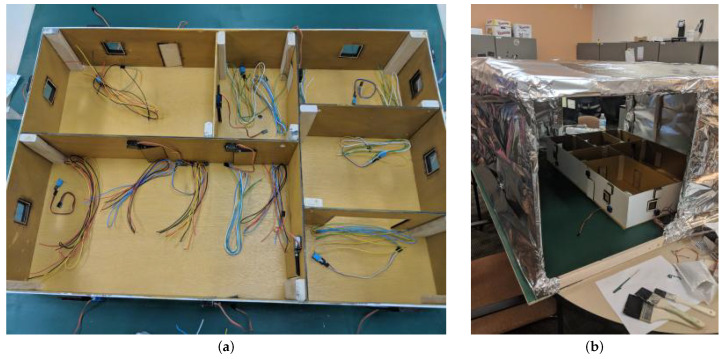
(**a**) The layout of the ScaledHome-2 testbed. (**b**) The testbed in the environmental enclosure.

The experimental data were collected using ScaledHome-2 [36,37], a 1:12 scale model of a suburban home intended to facilitate experiments that would be prohibitively expensive or even impossible to do with real-world homes. The initial ScaledHome was developed by Ling et al. [38]. The sensors of the testbed capture temperature, humidity, and light in every room, measure the energy consumption, and track the solar power generation and energy storage capabilities (see Figure 1).

The home models the architecture of a typical small suburban home in the Southeast US, with two bedrooms, a bathroom, a living room and a kitchen with an attached dining room. The prototype had been built from plywood, that had been cut using a laser cutter based on blueprints designed in SolidWorks. The implemented design for windows was a 2${}^{\prime \prime }$× 2${}^{\prime \prime }$ wooden frame with a centered 1.75${}^{\prime \prime }$× 1.75${}^{\prime \prime }$ hole, with a 2${}^{\prime \prime }$× 2${}^{\prime \prime }$ pane of acrylic glued onto it. Later, we added an additional layer of wood (1.75${}^{\prime \prime }$× 1.75${}^{\prime \prime }$ with a 1.5${}^{\prime \prime }$× 1.5${}^{\prime \prime }$ centered hole) to better fit into the wall. The implemented door design was a 4${}^{\prime \prime }$× 2${}^{\prime \prime }$ frame with an additional 3.75${}^{\prime \prime }$× 1.75${}^{\prime \prime }$ frame glued on. The additional frames added to doors and windows were added to counteract the distance the motors rest from their position on the walls. We used fifteen Adafruit micro servo motors as actuators; eight for windows and seven for doors.

Inside the house, we placed seven DHT11 temperature and humidity sensors: one in each room, and two in the living room and kitchen due to the size of the rooms. These sensors have a low power requirement and small size, allowing them to be inserted into the rooms of the scaled home. They provide an accuracy of ±5% in the humidity range of 20 to 80%, and ±2${\;}^{\circ }$C in the temperature range of 0–50${\;}^{\circ }$C. We balanced the positions of the sensors such that if there were multiple sensors in a room, they were placed on opposite sides of the room, otherwise each sensor was placed in the middle of its assigned room.

Two Raspberry Pi 3 devices were employed to collect data from the scaled home. One of them was used for collecting temperature and humidity information from the seven embedded sensors as well as switching the heat lamp and the fan on. The second Raspberry Pi controlled the fifteen motors for opening and closing of the doors and windows. All changes in motor and sensor status have been recorded. As the Raspberry Pi did not have enough power to handle the standing current and simultaneous running of all fifteen motors, we used an Adafruit Pi HAT, a module that adds an external power source to the Pi.

### 3.2. Data Acquisition Procedure

To learn the behavior of the system and verify the accuracy of the model, we need to run experiments to collect both training and test data. The most accurate model would be obtainable with exhaustive and repeated testing in a large span of situations. This is not possible in a real-world home: we cannot move a Florida home to Minnesota for the sake of experiments that involve extreme cold. Even for the ScaledHome testbed, where experiments are run at an accelerated timescale, it is not feasible to explore every combination, due to the very large number of sensor and actuator combinations. The system has 7 real-valued temperature readings that act as the initial state, with 15 different actuators inside the home, and 2 external actuators for the environmental model. This means a space of $2^{17}\times q^{7}$ different possible settings, where *q* is the level of quantization used for the temperature sensors. For instance, if we measure temperature with an accuracy of 1F, and consider a range of 60–120 F, then the number of possible states is $2^{17}\times 60^{7}=3.67\times 10^{17}$. Even with the significantly more accelerated data collection of the scaledhome, we cannot hope to acquire data for even a minor fraction of the possible configurations.

Fortunately, for the objective of this paper, we do not need to gather exhaustive experimental data; we only need to gather data that would be also feasible to collect in a real-world dataset. In our experiments, we collected data for eight simulated day/night cycles, modeling four summer days (with a longer day cycle) and four winter days (with a shorter day cycle). We used a variety of random actuator settings during these experiments. The temperature and humidity settings as well as the actuator states were collected every minute, with one minute of testbed time corresponding to approximately 20 min of real-world time. In total, we performed measurements at 1667 timesteps with 33 measurements at each timestep for a total of 55,011 data points. Half of this data was used for training and half as testing data.

## 4. Predictive Models in a Smart Home

### 4.1. Defining the Problem

A predictive model in our context is a parameterized function that takes as input the vectors of the actuators and measurements at current time *t* and returns the vector of predicted measurements at the next timestep:

(1) $$pred\left(m_{t},a_{t},\theta \right)\to {\hat{m}}_{t+1}$$

The error of a prediction (the loss function) is a distance function between the real and the predicted values. Throughout this paper, we will use the root mean squared error (RMSE) to measure the performance of the predictor. Let us consider a dataset $D=\{\ldots \left(m_{t},a_{t},m_{t+1}\right)\ldots \}$ of collected experimental data. The loss of the predictor over this dataset will be

(2) $$L_{\mathrm{RMSE}}\left(pred,\theta ,D\right)=\sqrt{\frac{1}{\left||D|\right|}\sum_{i}\left|\right|m_{t+1},pred\left(m_{t},a_{t}\right){\left|\right|}_{2}^{2}}$$

A convenient formulation of learning is empirical risk minimization, which involves choosing the θ that minimizes the set of losses over the training dataset:

(3) $$\theta_{opt}=\underset{\theta }{\mathrm{argmax}}L\left(pred,\theta ,D\right)$$

In general, the predictor pred is limited to a family of parameterized functions by the particular algorithm used. The nature of the parameter vector θ changes accordingly. For instance, if we are using a linear regressor, the function will be a linear function with a relatively small number of parameters. For a neural network regressor, the vector θ contains all the weights of a neural network, and thus it is correspondingly larger. For parameter-free machine learning models such as *k*-nearest neighbor classification, the θ is the empty set (An alternative way to think about the *k*-nearest neighbor model is that the θ is the entire training dataset.).

For our experiments, we will use a slight modification of this model, by predicting not the entire measurement vector $m_{t+1}$, but its individual components $m_{t+1}^{i}$ at a time. This effectively means that we are learning an individual predictor for each room in the house. The next question is whether the training of this model should still receive the full set of features $m_{t},a_{t}$ as input (which we will call a *large* set of features), or only a more limited subset. For instance, we can limit the data to only the room itself (a *minimal* feature set), or to the room and its immediate surroundings (a *medium* feature set). This choice has immediate practical importance because the smaller feature sets are significantly cheaper to acquire and the models easier to train.

### 4.2. Regression Algorithms

For our experiments, we considered a selection of regression algorithms from the machine learning literature appropriate for the problem and the available data. The algorithms included a simple parametric model (linear regression), parameter-free models (k-nearest neighbor regression), and more complex ensemble models (AdaBoost and Random Forest). We did not include deep neural network-based regression models in these experiments, as the large parameter space of neural networks would likely overfit on the limited training data. The algorithms and parametrizations used were as follows:

**Lin Regr:** This algorithm performs a linear regression over the features in the input. The predicted temperature change is expressed as a linear combination of the sensed input features. The optimization target of the algorithms is the minimization of the sum of the squared errors. In machine learning, linear regression is often used as the baseline predictor whenever we need to predict values based on features. In this case, we know that certain features of the system are not linear: for instance, whether the door to the outside is open or close affects the temperature differently when the temperature outside is higher or lower than the current temperature of the home. Nevertheless, we expect linear regression to capture many features of the system dynamics. Compared to more expressive predictive models, the advantage of linear regression is that it can be fully trained with a small amount of data.

**kNN *k* = 3** and **kNN *k* = 20:** *k*-nearest neighbor regression is a non-parametric model that predicts the value by finding the nearest *k* samples from the training data according to some metric. The returned value is a combination of the values associated with these *k* samples—in our experiments, we used a uniform average. Such models are often used when little prior information is known about the phenomena the regressor aims to model. For instance, *k*-nearest neighbor regressors can naturally handle nonlinear data.

The main challenges in using this model are the choice of the value of the *k* and the choice of the metric. We have used the Minkowski metric with the parameter p=2, making it equivalent to the standard euclidean distance. While a larger value of *k* is beneficial by lowering the effect of noise and outliers, as the dimensionality of the input space increases, higher *k* values will pick up sample points that might be farther and farther from the queried sample. We have run two sets of experiments with different values of *k*. The k=3 value is the smallest value that yields acceptable performance in a system, while k=20 was chosen as the largest value that is feasible given the size of our training data (if *k* is comparable to the size of the training data, kNN would return the global average independently of the input).

**RandomForest:** Random forest is an ensemble method that fits several classifying decision trees on random subset of the dataset. By averaging over the matching classifiers, the approach aims to improve the accuracy and control the overfitting. The main hyperparameters of this approach include the maximum number of trees, the maximum depth of the trees, and the criterion for splitting a branch in the tree. Through a set of preliminary experiments, we chose the number of trees to be 10 with a maximum depth of 2. We also used the mean squared error as a splitting criterion, which is aligned with our performance criteria.

**AdaBoost:** Like random forest, AdaBoost is an ensemble model, that aims to use a set of weak regressors (typically, decision trees) to solve a harder regression problem. AdaBoost fits regressors one by one, with subsequent regressors solving more difficult cases. In practice, AdaBoost is one of the most widely used algorithms as in many applications produces competitive results out-of-the-box, without the need for hyperparameter tuning. The default hyperparameters used were 50 weak estimators implemented as decision trees with a maximum depth of 3.

## 5. Experimental Results

### 5.1. Comparing the Prediction Algorithms with a Minimal Feature Set

In the first series of experiments, we assume a minimal set of features for the prediction algorithms: the previous state of the temperature in the given room and a boolean value that shows whether the heating lamp modeling the sun is on or off. In all the experiments in this section, the machine learning algorithms had been trained to predict the change in the temperature.

**Figure 2:**
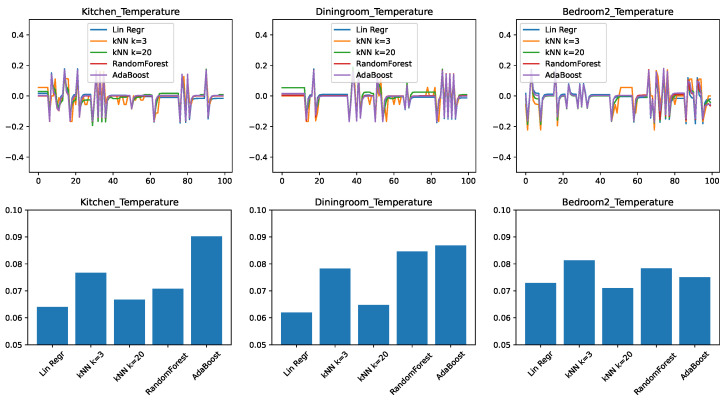
Comparing different regressors for temperature change prediction with a minimal feature set. (**top**) Prediction error in time. (**bottom**) RMSE over the test set.

Figure 2 shows the results for three representative rooms in the home: the kitchen, the dining room, and the second bedroom. These rooms had been chosen because, due to their location in the home and the size of the windows, the dining room shows the slowest change in response to external conditions, the bedroom is the fastest, with the kitchen having an intermediate position. Figure 2—top shows the prediction error, plotted in time for a representative 100-min interval from the test set. Depending on whether the predictor overestimated or underestimated the temperature, this error can be positive or negative, with a value of zero corresponding to no error. Figure 2—bottom shows the root mean squared error (RMSE) of the prediction, for the entire test data. This value is always positive, with a lower value being better.

We can draw the following observations from the results. None of the predictive models is perfectly able to predict the evolution in time: all models make errors both in the positive as well in the negative direction. We find that errors are correlated with each other in time. When one model shows an increased error in form of a spike, most of the time the other models will also show a spike in the same direction as well. This tells us that the errors are due to external circumstances that make the prediction generally harder to make.

To put the RMSE results in perspective, we need to consider that the prediction applies to changes in the temperature; while the individual errors for the span of one minute are small (lower than 0.1F), they can significantly add up over longer periods of time. The first observation is that the accuracy of the predictions is not the same across different rooms: here the dining room and the kitchen appear to be easier to predict, given the right predictor. We also find that linear regression performs very well, being clearly the best for the kitchen and dining room, and close to best in the case of the bedroom. The practical implication here is that in the case of a limited number of features available, linear regression, despite its simplicity, is a highly competitive approach.

We find that much more complex algorithms such as Random Forest and AdaBoost perform the worst in this set of experiments. Our conjecture is that the low dimensionality of the input limits the usefulness of the ensemble approaches, as the algorithms struggle to create sufficient diversity in the ensembles.

Another interesting observation can be made about the differently parameterized versions of the *k*-nearest neighbor algorithm. The versions with k=3 performed comparatively poorly, while the version with k=20 performed significantly better, being the best predictor for the bedroom. Our conjecture is that this is due to the noise in the data. By only looking for the 3 nearest matches, there is a high chance that the predictor picked up a sample with a high noise, which will be then propagated to the regression result. In contrast, with k=20 the noise is averaged out over a larger number of samples, making the prediction more accurate.

Our final conclusion from this set of experiments is that in the case of a minimal set of features, the appropriate choice of predictor is either linear regression or a *k*-nearest neighbor model with a relatively large value of *k*. Another conclusion, from the very different values we obtained for different rooms, is that predictor models must be learned on a home-by-home and room-by-room basis.

### 5.2. Comparing the Prediction Algorithms with a Medium Feature Set

In the second set of experiments, we repeated the experiments from the previous section with a larger feature set. In these experiments, in addition to the previous temperature settings and the status of the heating lamp, we added the temperature outside the scaled home and two boolean values: the first showing whether the given room’s windows and external doors are closed while the second one showing whether the rooms internal doors are closed.

**Figure 3:**
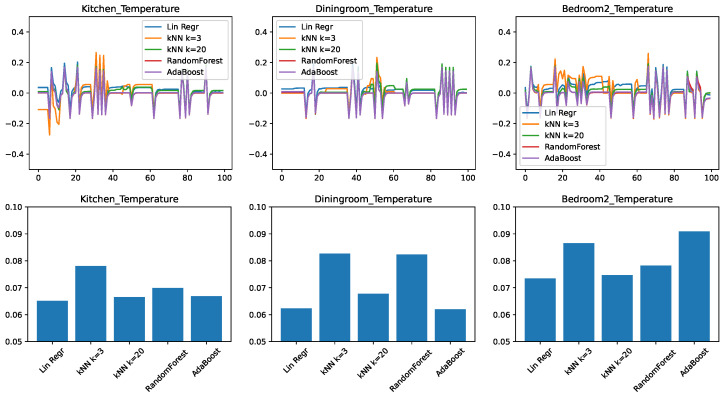
Comparing different regressors for temperature change prediction with a medium feature set. (**top**) Prediction error in time. (**bottom**) RMSE over the test set.

The results for the evolution of the error in time and the RMSEs are shown in Figure 3. We find that many of the observations from the minimal feature set case transfer to the medium feature set as well. The kitchen and the dining room remain better predictable with the right predictor. There is a consistent improvement with the *k*-nearest neighbor approach when raising the *k* value from 3 to 20.

The most striking difference is that with the increased number of features the ensemble approaches RandomForest and AdaBoost had significantly increased in their accuracy compared to the other approaches and now they are essentially competitive with linear regression and kNN k=20 for the kitchen and bedroom cases.

Nevertheless, from the point of view of practical implementation, even for this case, choosing linear regression or kNN k=20 is likely a better choice, because for equivalent performance that simpler algorithms are usually preferred.

### 5.3. Comparing the Prediction Algorithms with a Large Features Set

**Figure 4:**
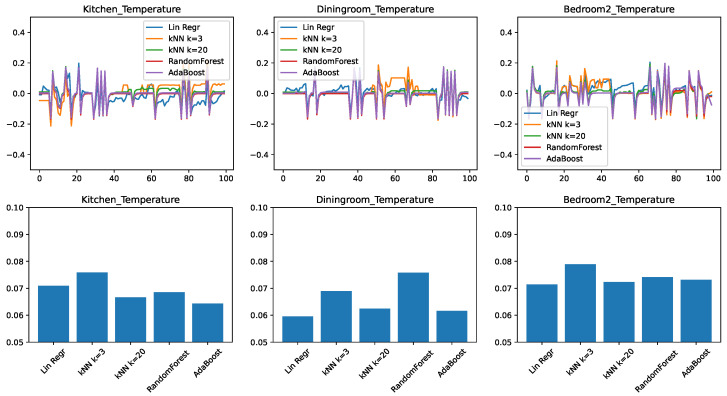
Comparing different regressors for temperature change prediction with a large feature set. (**top**) Prediction error in time. (**bottom**) RMSE over the test set.

In the final set of experiments, we trained and tested predictors on all the temperature-related features, we could obtain on the ScaledHome testbed. This includes previous values of temperatures both in the given room and other rooms, the outside temperature, and the open/closed status for every door and window in the home. Figure 4 shows the experimental results. We find that the trends that we observed when moving from the small feature set to the medium are continued here. The ensemble methods got even better with the larger feature set—in particular, AdaBoost is clearly the best for the kitchen prediction and very competitive in the other two cases. Linear regression, on the other hand, is still the winner for the prediction of the dining room. This is probably due to the same factors that make the dining room temperature change the slowest in response to external factors.

### 5.4. Comparing Different Feature Sets with Linear Regression

In the previous examples, we discussed how different regression models behave for a specific set of features. Now we take a different perspective by investigating how a given regressor performs for a given set of features.

Let us first discuss our expectations and what types of insights we aim to obtain from these investigations. First, our feature sets are successive supersets of each other: Minimal ⊂ Medium ⊂ Large. It appears, therefore, that in an ideal world, where the regressors would be able to efficiently take advantage of all the provided information, the large dataset would always provide a better result. In practice, however, this is not the case. Some data points in the large dataset might have a very remote relation to the investigated quantity—for instance, that door between the bedroom and bathroom might not affect the kitchen temperature in any measurable way. However, this extra information might bring in additional noise, which some algorithms might be more efficient in filtering out than others. Conversely, certain algorithms might be more likely to overfit the data when the dimensionality is low. Thus, our experiments aim to answer the question: (a) is more data always better? and (b) which algorithms are more suitable for taking advantage of more data?

**Figure 5:**
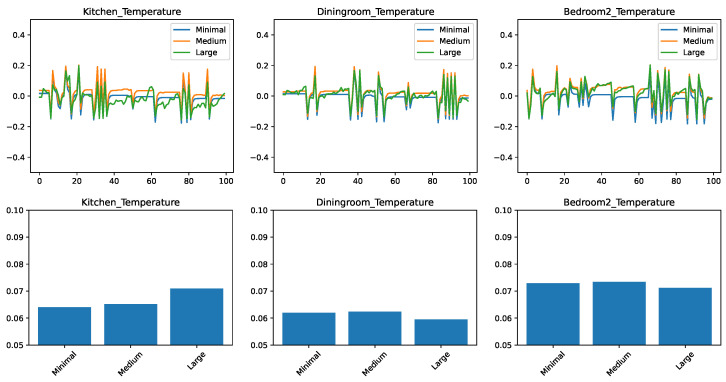
Comparing the prediction of a linear regressor for different feature sets. (**top**) Prediction error in time. (**bottom**) RMSE over the test set.

Figure 5 compares the performance of the linear regression algorithm on the dataset of different sizes. The format of the figure is the same as in our previous experiments, with the time-based error on the top and RMSE at the bottom. The RMSE bar chart shows that, in general, the linear regressor is not able to take advantage of the additional data. In fact, for the case of the kitchen predictor, the more data we have, the worse the prediction accuracy.

The time-based evolution of the error shows us more about the reason for this behavior. For the prediction based on the minimal feature set, there are periods where the error is nearly zero. The error is mostly concentrated in the spikes. From the correlation of the spikes between different predictors, we conjecture that they correspond to points in time when the temperature is more difficult to predict. However, when the linear predictor was trained on the medium and large datasets, the stretches which were previously of zero error show a clear drift away from the correct value. We conjecture that this is caused by the linear regressor not being able to ignore irrelevant information from the larger datasets.

**Figure 6:**
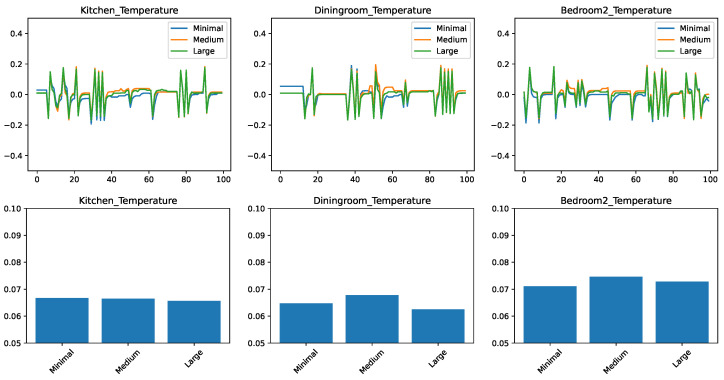
Comparing the prediction of a *k*-nearest neighbor regressor with k=20 for different feature sets. (**top**) Prediction error in time. (**bottom**) RMSE over the test set.

Figure 6, which performs the same study for the *k*-nearest neighbor predictor with k=20, shows a different picture. For the kitchen and dining room cases, the large datasets lead to the best performance, and it is also very competitive for the bedroom prediction. On the other hand, the medium dataset was close to the worst in most cases. This shows us that the *k*-nearest neighbor regression, at least in its standard, unweighted format, is very sensitive to spurious information in the dataset. The algorithm relies on the proximity of the queried point to points in the training data. However, two points might be close in features that have a minor role in the prediction and far away in the important features. A distance function with custom weights can solve this problem, but it opens the new problem of finding the weights, which require significant human intervention.

**Figure 7:**
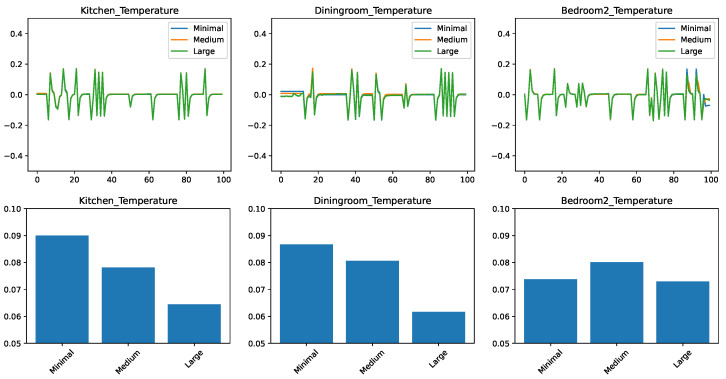
Comparing the prediction of the AdaBoost regressor for different feature sets. (**top**) Prediction error in time. (**bottom**) RMSE over the test set.

Finally, the results for the AdaBoost regressor are shown in Figure 7. The results clearly show that the AdaBoost regressor can take advantage of extra data—in fact, for the kitchen and dining room case, there is a very pronounced improvement from the minimal to medium to large feature sets. For the bedroom, the medium feature set is an outlier, showing worse results.

We conclude that if there is a sufficiently large number of features, then the boosting algorithm can select the relevant features and provide high accuracy. However, the results are not as good for a smaller number of features.

Let us now answer the questions we pose at the beginning of this section. Is more data, such as more features, better? The answer is yes, provided that the machine learning algorithm provides appropriate support for it. The overall best predictions had been obtained with the large feature set and AdaBoost. The other machine learning algorithms we considered, however, were not able to consistently take advantage of the large feature sets. Conversely, we found that if our feature sets are limited, linear regression yields the best prediction; more complex algorithms are not justified for limited data.

## 6. Conclusions

In this paper, we described a series of experiments performed on a scaled model of a real-world home. We used the collected data to build inputs to machine learning models that learn to predict the temperatures of individual rooms. We experimented with inputs with different sets of features, from a minimal set of features only collected from the given room to a medium set that adds the immediate environment and the large set that considers data from the entire home. The objective of our study was to investigate whether the individual room temperatures are predictable using specific machine learning techniques and specific sets of features.

Our findings show that, in general, the temperatures are predictable with reasonable accuracy and larger, holistic datasets can improve the prediction. However, our experiments also uncovered several unexpected conclusions. We found that different rooms in the house require not only different models, but also differ in the predictability of the temperatures. Second, we found that different machine learning algorithms behave differently function of the available data. Linear regression is the best performing algorithm for a small number of features, while AdaBoost performs best for the large feature set. We found that for the ScaledHome scenarios we considered, *k*-nearest neighbor regression requires a relatively large number for the *k*-value to smooth over the sensor noise. Furthermore, *k*-nearest neighbor is sensitive to irrelevant features in the feature set and requires more careful feature engineering compared to other algorithms.

Let us now consider the applicability, advantages, and disadvantages of the proposed method. The approach we propose allows a user to check whether a machine learning model predicting temperature and other values can be trained for a given home configuration and set of sensors. An advantage of the scaled physical model is that it can be built and experimented with without requiring expertise in building thermodynamics, allowing the capture of important aspects such as the dependent and independent variables of the model. A disadvantage of the approach is that the model will not be sufficiently accurate for the learned model to be directly transferred to a home. The approach can provide the choice of the architecture and parametrization of the machine learning model to be used—but the model itself will need to be retrained with real-world data. An implication of this is that the approach is best applicable to suburban, single-family homes. Whenever a construction project can budget a sufficient amount of expert modeler person-hours for an accurate model, a high-precision simulator might be a better choice.

Our future work will focus on applying the lessons of these predictive studies to develop an active, energy-efficient control system and validate it on the ScaledHome testbed.

## Data Availability

Not applicable.
